# Editorial: Understanding and bridging the gap between neuromorphic computing and machine learning, volume II

**DOI:** 10.3389/fncom.2024.1455530

**Published:** 2024-10-03

**Authors:** Lei Deng, Huajin Tang, Kaushik Roy

**Affiliations:** ^1^Department of Precision Instrument, Center for Brain Inspired Computing Research, Tsinghua University, Beijing, China; ^2^College of Computer Science and Technology, The State Key Lab of Brain-Machine Intelligence, Zhejiang University, Hangzhou, China; ^3^MOE Frontier Science Center for Brain Science and Brain-Machine Integration, Zhejiang University, Hangzhou, China; ^4^Department of Electrical and Computer Engineering, Purdue University, West Lafayette, IN, United States

**Keywords:** spiking neural networks, neuromorphic computing, neuromorphic hardware, artificial neural networks, machine learning

## Introduction

Pursuing intelligence is a long-term goal of the human, toward which two routes have been paved on the road: neuromorphic computing driven by neuroscience and machine learning driven by computer science (Pei et al., 2019). Spiking neural networks (SNNs) and neuromorphic chips (Basu et al., 2022; Christensen et al., 2022) dominate the neuromorphic computing domain, while artificial neural networks (ANNs) and machine learning accelerators (Deng et al., 2020) dominate the machine learning domain. Neuromorphic computing with efficient models and hardware has shown energy efficiency superiority (Renner et al., 2021), however, still lies in its infant stage and presents a gap in terms of accuracy and applications compared to the mature machine learning ecosystem.

To this end, we proposed a Research Topic, named “*Understanding and bridging the gap between neuromorphic computing and machine learning*,” in Frontiers in Neuroscience and Frontiers in Computational Neuroscience in 2019, and have successfully published 14 articles on neuromorphic computing and machine learning (Deng et al., 2021). Encouraged by such positive impetus for the neuromorphic computing community, we relaunched the Research Topic in 2022. This time, we have accepted 11 submissions in the end. The scope of these works covers neuromorphic models and algorithms, hardware implementation, and programming frameworks.

## Neuromorphic models and algorithms

SNNs encode information in spike events and process information using neural dynamics, which differ from ANNs. Due to the complicated spatiotemporal dynamics and non-differentiable spike activities, the SNN domain uses plasticity-based unsupervised learning algorithms (Diehl and Cook, 2015) for a long time but suffers from low accuracy. To break the bottleneck of lacking effective learning algorithms, the sophisticated backpropagation method in machine learning has been introduced into SNNs (Lee et al., 2016; Wu et al., 2018), which greatly improves the performance of SNNs and thus extending the scope of neuromorphic models and applications (Yao et al., 2023). A comprehensive survey of the direct learning-based deep SNNs can be found in the review article from Guo et al., mainly categorized into accuracy improvement methods, efficiency improvement methods, and temporal dynamics utilization methods. Besides this review article, we accepted six articles on neuromorphic models and algorithms in this Research Topic, which are briefly summarized below.

With the extensive researches on SNN learning algorithms, how to combine the complementary advantages of bio-plausible unsupervised learning and powerful supervised learning is becoming an emerging and interesting Research Topic (Wu et al., 2022). Some learning rules for SNNs adopt a three-factor Hebbian update: a global error signal modulates local synaptic updates within each layer. Unfortunately, the global signal for a given layer requires processing multiple samples concurrently, but the brain only sees a single sample at a time. Daruwalla and Lipasti propose a new three-factor update rule where the global signal correctly captures information across samples via an auxiliary memory network. The auxiliary memory network can be trained a priori independently of the dataset being used with the primary network. This work presents an explicit connection between working memory and synaptic updates.

Most learning methods only consider the plasticity of synaptic weights. To improve the learning performance and biological plausibility, Wang proposes a new supervised learning algorithm for SNNs based on the typical SpikeProp method, in which both the synaptic weights and delays are adjustable parameters. Sun et al. further combine learnable delays, local skip-connections and an auxiliary loss term to enhance the accuracy and stability of SNNs, which is validated on spoken word recognition benchmarks.

Current SNNs can only focus on information within a short time period, which makes it difficult for them to make effective decisions based on global information. Chen et al. propose SNNs with working memory (SNNWM) to handle spike trains segment by segment, inspired by recent neuroscience advances. This model can help SNNs obtain global information and reduce the information redundancy between adjacent time steps. To better leverage the temporal potential of SNNs, Wu X. et al. propose a self-attention-based temporal-channel joint attention SNN (STCA-SNN) with end-to-end training by inferring attention weights along both temporal and channel dimensions concurrently. This method can model global temporal and channel information correlations, enabling the network to learn “what” and “when” to attend simultaneously.

High energy efficiency is a well-known advantage of SNNs, which is tightly associated with the sparse spike activities. To reduce the redundant spike counts, Fois and Girau propose weight-temporally coded representation learning (W-TCRL), which utilizes temporally coded inputs and leads to lower spike counts and improved energy efficiency. Furthermore, they introduce a novel spike-timing-dependent plasticity (STDP) learning rule for stable learning of relative latencies within the synaptic weight distribution. This work improves the image reconstruction error while achieving significantly higher sparsity in spike activities.

## Hardware implementation

Neuromorphic models enjoy low computational costs owing to the binary spike representation and sparse operations. However, directly executing SNNs on GPUs without tailored optimization is inefficient. Neuromorphic hardware is designed for the efficient execution of SNNs via event-driven computing (Merolla et al., 2014). In this Research Topic, we accepted three articles on hardware implementation of SNNs in this Research Topic.

Probabilistic sampling is an effective approach for making SNNs achieve Bayesian inference, but also a time-consuming operation on conventional computing architectures. To address this problem, Li et al. design a specific accelerator on FPGA to improve the execution of SNN sampling models by parallelization. The streaming pipelining and array partitioning operations are used to achieve acceleration with the lowest resource consumption. The Python productivity for Zynq (PYNQ) framework is combined to efficiently migrate models onto FPGA. This work promises implementing complex probabilistic model inference in embedded systems.

Huang et al. propose the MAC array for the acceleration of SNN inference, which is a parallel architecture on each processing element of SpiNNaker 2 (Liu et al., 2018). The authors further investigate the parallel acceleration algorithms for collaborating with multi-core MAC arrays. The proposed Echelon Reorder model information densification algorithm can achieve efficient spatiotemporal load balancing and optimization performance with the help of the adapted multi-core two-stage splitting and authorization deployment strategies. This work expands the application scope of the general sparse matrix-matrix multiplication (SpGEMM) issue to SNNs.

The decentralized manycore architecture with high computing parallelism and memory locality is widely adopted by neuromorphic chips. However, its fragmented memories and decentralized execution lowers the resource utilization and processing efficiency. Wang et al. propose the mapping limit concept which points out the resource saving upper limit during logical and physical mapping when deploying neural networks onto neuromorphic chips. A closed-loop mapping strategy with an asynchronous 4D model partition for logical mapping and a Hamilton loop algorithm (HLA) for physical mapping are elaborated. Their methods and performance gains are validated on the TianjicX neuromorphic chip (Ma et al., 2022), which is helpful for building a general and efficient mapping framework for neuromorphic hardware.

## Programming frameworks

Software is one of the key components in the ecosystem of neuromorphic computing (Fang et al., 2023), which is sometimes more important than the hardware itself because it determines how much practical efficiency we can gain from the peak efficiency of hardware. In this Research Topic, we accepted one article on the programming framework for neuromorphic models in this Research Topic.

Wu Z. et al. introduce a user-friendly brain-inspired deep learning (BIDL) framework for generalized and lightweight spatiotemporal processing (STP). Researchers can use the framework to construct deep neural networks which leverage neural dynamics for processing spatiotemporal information and ensure high accuracy. The framework is compatible for various types of spatiotemporal data such as videos, dynamic vision sensor (DVS) signals, 3D medical images, and natural languages. Moreover, BIDL incorporates several optimizations such as iteration representation, state-aware computational graph, and built-in neural functions for easy deployment on GPUs and neuromorphic chips. By facilitating the exploration of different neural models and enabling global-local co-learning, BIDL shows potential to drive future advancements in bio-inspired research.

## Conclusion

Neuromorphic computing is a neuroscience-driven domain in pursuing brain-like intelligence, which is an important route distinct from machine learning. Although neuromorphic systems have not yet demonstrated superior performance over machine learning systems in main stream intelligent tasks, we believe it can be significantly improved when the neuromorphic ecosystem is constructed and becomes iterative between algorithms, models, hardware, software, and benchmarks. This Research Topic is a quite minor step. We hope future works can really bridge the gap between neuromorphic computing and machine learning, along the way to reach the long-term goal of mimicking brain intelligence.

## Data Availability

The original contributions presented in the study are included in the article/supplementary material, further inquiries can be directed to the corresponding author.
